# Analysis of* In Vitro* Cyto- and Genotoxicity of Barbatimão Extract on Human Keratinocytes and Fibroblasts

**DOI:** 10.1155/2018/1942451

**Published:** 2018-10-08

**Authors:** Neida L. Pellenz, Fernanda Barbisan, Veronica F. Azzolin, Thiago Duarte, Aline Bolignon, Moisés H. Mastella, Cibele F. Teixeira, Euler E. Ribeiro, Ivana B. Mânica da Cruz, Marta M. M. F. Duarte

**Affiliations:** ^1^Postgraduate Program of Pharmacology, Federal University of Santa Maria, Santa Maria, RS, Brazil; ^2^Postgraduate Program of Gerontology, Federal University of Santa Maria, Santa Maria, RS, Brazil; ^3^Phytochemical Research Laboratory, Department of Industrial Pharmacy, Federal University of Santa Maria, Santa Maria, RS, Brazil; ^4^Third Age Open University, University of Amazonas State, Manaus, AM, Brazil; ^5^Brazilian Lutheran University (ULBRA), Santa Maria, RS, Brazil

## Abstract

Barbatimão (*Stryphnodendron adstringens*, Mart.) is a native Brazilian species used in traditional medicine and some commercial preparations owing to its strong wound-healing activity. However, controversy regarding its use due to safety concerns over the potential genotoxic effect of this plant remains. In order to clarify this issue, the effect of hydroalcoholic extract of barbatimão* in vitro* on cell viability, DNA damage, and induction of apoptosis in two commercial cell lines of keratinocytes (HaCaT) and fibroblasts (HFF-1) was evaluated. Barbatimão stem bark hydroalcoholic extract (70% ethanol) was obtained and lyophilized for subsequent use in all experiments. The main bioactive molecules quantified by HPLC were gallic acid, caffeic acid, quercetin, catechin, and epigallocatechin gallate (EGCG). Barbatimão (0.024 to 1.99 mg/mL) was found to decrease cellular mortality as compared to the control group. GEMO assay, a noncellular DNA protocol that uses H_2_O_2_-exposed calf thymus DNA, revealed not only a genotoxic effect of barbatimão, but also a potential genoprotective action against H_2_O_2_-triggered DNA fragmentation_._ These results indicated that barbatimão at concentrations of 0.49 and 0.99 mg/mL, which are near to the levels found in commercial preparations, exerted an* in vitro* genoprotective effect on cells by decreasing the levels of DNA oxidation quantified by 8-hydroxy-2′-deoxyguanosine (8-OHdG) and reactive oxygen species (ROS) levels. Gene and protein apoptotic markers, quantified by qRT-PCR (*BAX/Bcl-2* genes) and immunoassays (Caspases 3 and 8), respectively, also indicated a decrease in apoptotic events in comparison with control cells. Collectively, the results suggest that barbatimão could exert genoprotective and antiapoptotic effects on human keratinocytes and fibroblasts.

## 1. Introduction

Many studies have described potential effectiveness of phenolic compounds in medicine due to their antioxidant, anti-inflammatory, antimicrobial, and proliferative properties [1, 2]. Some of these polyphenol molecules have been described in plants such as* Stryphnodendron adstringens* (Mart.), a Brazilian species popularly known as “barbatimão,” with strong wound-healing activity [3, 4]. Barbatimão is a native species of the Savanah Biomes (Cerrado and Caatinga), where a bark extract is used by traditional communities as a wound-healing natural product [5–9]. Prior investigations have described potential causal mechanisms associated with the healing efficacy of barbatimão, such as antioxidant, anti-inflammatory, and antimicrobial activities [6, 10–13].

Considering that barbatimão is effective and inexpensive, this bark extract has been included in some commercial Brazilian medicines. Its inclusion as a phytotherapeutic plant is also based on complementary studies that have described low acute and chronic toxicity in barbatimão experimental models [14–17].

However, there are some aspects of the plant that need to be studied for the safe use of barbatimão as a drug, such as its cito- and genotoxic potential. Therefore, it is necessary to perform complementary investigations to evaluate the effect of barbatimão on human cellular DNA damage modulation. The present study evaluated the modulation of genotoxicity and apoptosis as an indicator of the effect of a commercial barbatimão extract on DNA damage in human keratinocytes and dermal fibroblast commercial cell lines.

## 2. Materials and Methods

### 2.1. Reagents

Chemical reagents including acetonitrile, formic acid (purity > 98%), gallic acid (purity > 98%), and caffeic acid (purity > 98%) were purchased from Merck (Darmstadt, FRM, Germany), and quercetin (purity > 98%), catechin (purity > 98%), rutin (purity > 98%), and kaempferol (purity > 98%) used as reference molecules were obtained from Sigma (Saint Louis, MI, United States). High-pressure liquid chromatography with a diode array detector (HPLC-DAD) was performed using Shimadzu Prominence Auto Sampler (SIL-20A) high-pressure liquid chromatography (HPLC) system (Shimadzu, Kyoto, Japan), equipped with Shimadzu LC-20AT reciprocating pumps connected to a DGU 20A5 degasser with a CBM 20A integrator, SPD-M20A diode array detector, and LC solution 1.22 SP1 software. Materials used in cell culture were purchased from Vitrocell Embriolife (Campinas, SP, Brazil) and Gibco-Life Technologies (Carlsbad, CA, United States). Molecular biology reagents were obtained from QIAGEN (Hilden, NW, Germany), Invitrogen (Carlsbad, CA, United States), and Bio-Rad Laboratories (Hercules, CA, United States).

Biochemical reagent protocols for performing the spectrophotometry assays were obtained from Sigma-Aldrich (St. Louis, MI, United States). The apoptotic and genotoxic markers marked by an ELISA immunoassay kit were obtained from Abcam (Cambridge, MA, United States). The equipment used included a SpectraMax i3x Multimode microplate reader (Molecular Devices, Sunnyvale, CA, United States) and Rotor-Gene Q 5plex HRM System (QIAGEN biotechnology, Hilden, NW, Germany).

### 2.2. Good In Vitro Methods Practices and Experimental Design

The* in vitro* protocols performed in the present investigation are according to presumptions described in OECD Guidelines for the Testing of Chemicals and in a draft of guidance document on Good* In vitro* Methods Practices (GIVIMP) organized by Griesinger [18]. The followed standard procedures were used in the present study to guarantee high data quality: (1) reagents and plastics of high quality and origin were used; (2) ATCC commercial lineages and standardized conditions described by the ATCC were used; (3) all experiments were initialized with cells at of 1 × 10^5^ concentration; (4) all experiments were carried out at controlled culture times identified in the results and/figures; (5) experiments conducted in 96-well plates were just in the internal wells in order to attenuate problems related to the evaporation of the medium which can cause large intravariation of the data; (6) in the 96-well plates each treatment was repeated at least five times; (7) all experiments were replicated at different moments, at least three times. In each replication, at least five repetitions of each treatment were tested; (8) details of data analysis used in GIVIMP were also followed and are presented in statistical section of this study.

**Figure 1:**
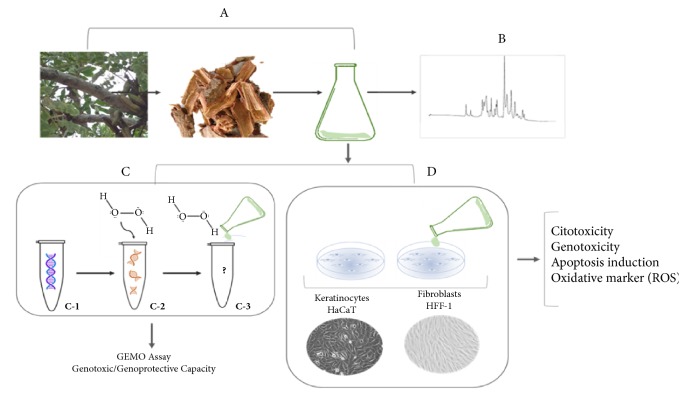
General experiment design. (A) Initially was obtained a hydroalcoholic extract using barbatimão bark samples. (B) Main bioactive compounds were quantified by HPLC-DA. (C) An exploratory noncelular GEMO assay was conducted to determine if barbatimão at 10 different concentrations could present some genotoxic or genoprotective capacity of the extract. (D) The in vitro protocols were performed in two commercial human cell lines of keratinocytes (HaCaT) and dermal fibroblasts (HFF-1). Additional protocols were conducted to evaluate barbatimão effects on DNA oxidation by quantification of DNA-8-OhdG levels and by intrinsic or extrinsic apoptosis induction by quantification and comparison with negative control group of gene BAX/Bcl-2 ratio and CASP 3 and 8 protein levels. Details of experimental design and assays used in this study are presented in Methods section.

General analysis performed in the present investigation is synthetized in Figure 1. Further, details of assays used in the present study will be detailed. Initially, to test potential toxicological effect of barbatimão on human cell lines, a hydroalcoholic extract using bark samples of this plant collected in Manaus, Amazonas State, Brazil (-3.10719S, -60.0261 3° 6′ 26′′W), and voucher specimens were deposited at the Herbarium of the Biological Sciences Course, Federal University of Santa Maria, RS, Brazil (Figure 1(A)). Main bioactive molecules present in the barbatimão extract were quantified by HPLC-DAD analyses (Figure 1(B)).

**Figure 2:**
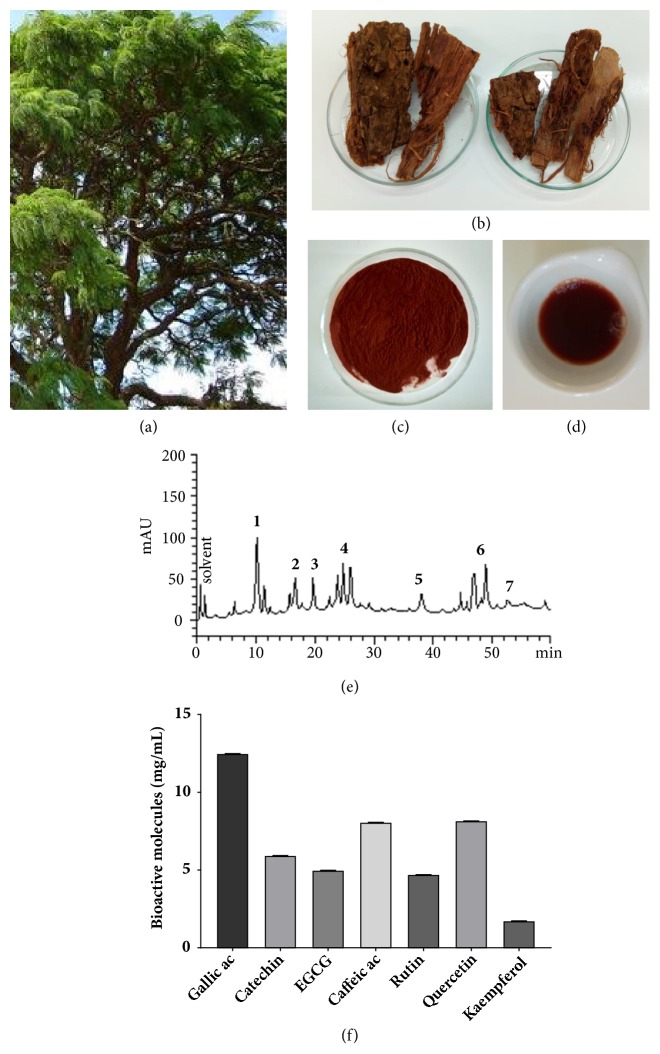
Barbatimão (*Stryphnodendron adstringens*, Mart.) components and measurement of bioactive molecules: (a) barbatimão tree; (b) stem bark used to produce barbatimão extract; (c) lyophilized barbatimão extract; (d) solution of hydroalcoholic extract of barbatimão used to treat cell cultures; and (e) representative HPLC profile of barbatimão hydroalcoholic extract. Gallic acid (peak 1), catechin (peak 2), EGCG (peak 3), caffeic acid (peak 4), rutin (peak 5), quercetin (peak 6), kaempferol (peak 7); (f) graphical representation of bioactive molecule quantification detected in barbatimão HPLC analysis.

**Figure 3:**
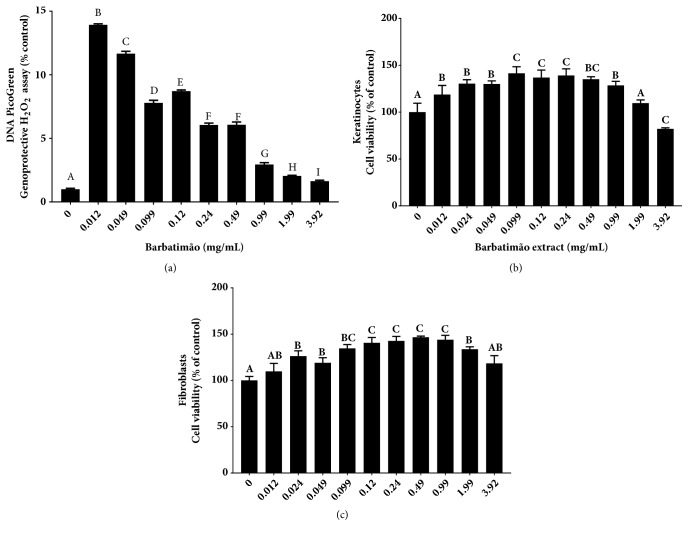
Barbatimão preliminary assays: (a) genoprotective capacity determined by GEMO noncellular assay that uses DNA PicoGreen® fluorescent dye (Cadoná et al., 2014). This dye presents specific affinity to binding with double-strand (ds) DNA levels. In GEMO assay a solution containing isolated calf dsDNA and H_2_O_2_ (1M) is produced. The H_2_O_2_ trigger extensive DNA fragmentation that causes decreasing in the fluorescence and here is considered the control group represented by 0 value. Cell culture supplementation with barbatimão hydroalcoholic extract at different concentrations showed significant increase in the fluorescence indicating genoprotective effect of this extract; barbatimão's viability effect on (b) keratinocytes and (c) fibroblast 24 h cell cultures measured by MTT-assay. Data were analyzed by ANOVA one-way analysis of variance followed by Tukey* post hoc* test and all statistical tests with p ≤ 0.05 were considered significant. Statistical differences among treatments were identified by different alphabetical letters, whereas same letters indicated no differences between each treatment compared to the others.

Before* in vitro* analyses, the noncellular GEMO assay was performed to identify potential genotoxic and/or genoprotective barbatimãos's capacity according to different concentrations (Figure 1(C)). The principle of this assay previously developed and validated by Cadoná et al. [19] is to verify the effect of some extract-test on fragmentation of double-strand (ds) DNA molecule that is concomitantly exposed to H_2_O_2_, an oxidative molecule. Therefore, if an extract-test has genotoxic capacity, dsDNA fragmentation triggered by H_2_O_2_ exposure will be more intense than negative control group with just dsDNA in the solution. On the contrary, if an extract-test has a genoprotective capacity, dsDNA fragmentation will be less intense than positive control group with dsDNA plus H_2_O_2_. GEMO assay is performed using a highly specific dsDNA dye (PicoGreen®), as a basic reagent. This dye is an ultrasensitive fluorescent reagent that allows quantification in the solution, just dsDNA, but not single DNA molecules and nucleotides. Therefore, high dsDNA concentration is associated to high fluorescence levels quantified by fluorimeter (Figure 1(C-1)). When dsDNA is fragmented by the presence of oxidant molecules, such as H_2_O_2_, fluorescence drops in comparison with negative non-H_2_O_2_ exposed control (Figure 1(C-2)). Therefore, the effect of some extract-test on this reaction can indicate its genotoxic or genoprotective effect. Genoprotective capacity is detected when fluorescence increases significantly in a solution containing dsDNA, H_2_O_2,_ and extract-test in comparison with a solution containing just dsDNA plus H_2_O_2_. On the contrary, if fluorescence decreases significantly with addition of extract-test in the same solution, this result indicates genotoxic capacity of the extract. Therefore, this assay was used as preliminary indication of genoprotective and/or genotoxic (Figure 1(C-3)) capacity of barbatimão extract at different concentrations.

All* in vitro* protocols were performed using two cell commercial lines of keratinocytes (HaCaT) and dermal fibroblasts (HFF-1) in 24 h cell cultures (Figure 1(D)). First protocol determined if similar concentrations tested in the GEMO assay could present some cytotoxic effects on these cell lines. Therefore, cell cultures were supplemented with barbatimão at 10 different concentrations (0.012, 0.024, 0.049, 0.099, 0.12, 0.24, 0.49, 0.99, 1.99, and 3.92). From these results, two barbatimão concentrations estimated to be found in Brazilian commercial wound-healing barbatimão products described in pharmaceutical package were used in the complementary protocols (0.49 and 0.99 mg/mL).

These protocols evaluated if barbatimão extract could induce cellular* in vitro* genotoxicity by quantification of 8-deoxyguanosine (DNA-8-OhdG) levels. This molecule is formed when DNA is oxidized and is considered a good biomarker of oxidative stress and oxidative DNA damage [20]. Extensive cellular and DNA damage can trigger apoptotic events on the cells. The intrinsic apoptotic events are triggered by increase of p53 protein levels, which are able to detect no-repaired DNA lesions and induce overexpression of Bcl-2-associated X protein (BAX gene), an apoptotic regulator. On the contrary, p53 protein induces downregulation of B-cell lymphoma 2 (Bcl-2) that plays a crucial role in promoting cellular survival and proliferation. Therefore, BAX/Bcl-2 ratio has been used as marker of intrinsic apoptosis events (when ratio is ≥ 1.0) in a large number of studies, such as performed by Bergandi et al. [21]. Upon induction of apoptosis, BAX becomes organelle membrane associated, and, in particular, mitochondrial membrane that becomes permeabilized releases cytochrome C into cytosol. The elevated cytochrome C concentration in the cytoplasm triggers caspases (CASP) pathway (including CASP 3 and 8) that regulate further apoptotic events. Moreover, caspases pathway can be triggered by an extrinsic pathway related to binding of some molecules with dead receptors that are present in the outside of cellular membrane. In this context, concomitant quantification of BAX/Bcl-2 gene expression ratio and CASP 3 and 8 proteins can be considered informative if some extract or product triggers apoptotic events and if events involve intrinsic or extrinsic apoptosis pathways. This protocol of apoptosis induction has been used in previous studies performed by our research group [22, 23].

As barbatimão extract can be clinically tested in several days, a final protocol was performed to evaluate if the chronic cell culture exposure to babartimão could present elevation of reactive oxygen species (ROS) levels and DNA 8-OHdG levels, which indicates DNA damage measure in 1, 3, and 5 days of cultures.

### 2.3. Barbatimão Extract Obtention and HPLC-DAD Procedures

Barbatimão hydroalcoholic extract was obtained in a manner similar to that described by Betoni et al. [24]. Barbatimão bark was dried, ground, and extracted with 70% ethanol at 4–8°C then filtered after 48 h. Filtration was done using Whatman No. 1 paper and the solvent removed using a rotary evaporator at reduced pressure, 45°C at 115 rpm. The resulting dry extract was obtained by lyophilization and stored at 20°C in a sterile flask until use. Quantification of the main bioactive molecules in the barbatimão stem bark extract was performed as previously described by Da Silva et al. [25]. Barbatimão hydroalcoholic extract at a concentration of 12 mg/mL was injected in a model SIL-20A Shimadzu Auto sampler. Separations were carried out using Phenomenex C18 column (4.6 mm × 250 mm × 5 *μ*m particle size). The mobile phase consisted of water with 1% formic acid (v/v) (solvent A) and HPLC grade acetonitrile (solvent B) at a flow rate of 0.6 mL/min and injection volume 40 *μ*L. The composition gradient was 5% solvent B reaching 15% at 10 min; 30% solvent B at 20 min; 65% solvent B at 30 min; and 98% solvent B at 40 min, followed by 50 min at isocratic elution until 55 min. At 60 min, the gradient reached the initial conditions again, following the method described by Da Silva et al. [25] with small modifications. The sample and mobile phase were filtered through a 0.45 *μ*m membrane filter (Millipore) and then degassed by ultrasonic bath prior to use. Stock solutions of standards references were prepared in acetonitrile at a concentration range of 0.030–0.500 mg/mL. Quantifications were carried out by integration of the peaks using the external standard method, at 254 nm for gallic acid, 280 nm for catechin, 327 nm for caffeic acid, and 366 nm for quercetin, rutin, and kaempferol. The chromatography peaks were confirmed by comparing retention time with those of reference standards and by DAD spectra (200 to 700 nm). All chromatography operations were carried out at ambient temperature and in triplicate.

### 2.4. Cell Culture Conditions and Treatments

The* in vitro* investigation used two commercial cell lines: immortalized human keratinocytes (HaCaT) and neonatal foreskin human dermal fibroblasts (HFF-1) obtained from American Type Culture Collection (ATCC). Cell cultures were reared in controlled conditions with Dulbecco's modified Eagle medium (DMEM) culture medium supplemented with 15% fetal bovine serum, 100 IU/mL penicillin, and 100 *μ*g/mL streptomycin. Cells were maintained at 37°C with 5% CO_2_ and 95% humidified atmosphere for 24 h. Potential cytotoxic effects of hydroalcoholic barbatimão extract on keratinocytes and fibroblasts were tested using ten different concentrations. The curve concentration of barbatimão analyzed here was based on a prior study by Costa et al. [15] and was used as a reference (0.012 to 3.92 mg/mL). To perform this curve, barbatimão lyophilized extract was diluted in culture medium and filtered using Whatman filter paper (2 *μ*) to prevent the presence of microorganisms in the solution.

Two barbatimão concentrations near to those found in commercial preparations of barbatimão extract were used to perform complementary analysis (0.49 and 0.99 mg/mL) in keratinocyte and fibroblast 24 h cell cultures. In cells exposed to these concentrations, DNA oxidative damage was evaluated by quantification of 8-hydroxy-2′-deoxyguanosine (8-OHdG) that is considered a biomarker for oxidative DNA markers and has been widely used in* in vivo* and* in vitro* studies [20]. We also evaluated potential modulation of apoptosis by barbatimão exposure via analysis of expression of two genes:* Bcl-2* (B-cell lymphoma 2) and* BAX* (bcl-2-like protein 4).* Bcl-2* gene is an inductor of cellular proliferation whereas* BAX* is an inductor of apoptotic events. Generally,* BAX/Bcl-2* gene expression ratio is used to detect if cell cultures are in apoptotic or proliferative processes [26]. In this case, values > 1 indicate an apoptotic state and potential genotoxic effect of barbatimão on cells. Quantification of two caspase (CASP 3 and 8) protein levels was also performed to confirm the influence of barbatimão on apoptotic processes. Methods used to determine gene expression and protein quantification involved qRT-PCR and immunoassay analysis are further described in more details below.

### 2.5. GEMO Assay

Before testing the potential effect of barbatimão on the DNA of keratinocytes and fibroblasts, a GEMO assay was performed as previously described by Cadoná et al. [19], which used Quant-IT™ PicoGreen® DNA kit (Invitrogen Life Technologies, SP, Brazil). GEMO is a noncellular protocol previously described by Cadoná et al. [19] that uses exposure of calf-DNA to H_2_O_2_. This molecule is genotoxic, increasing DNA fragmentation that is detected by PicoGreen dye. If a chemical molecule in the plant extract has a genoprotective effect, DNA fragmentation will be attenuated, and fluorescence in the test group will decrease in relation to the positive control group (obtained by exposure of calf-DNA to H_2_O_2_). A 96-well plate was filled with 10 *μ*L of calf thymus DNA (1 *μ*g/mL plus 70 *μ*L of TE buffer) containing varying barbatimão concentrations and 70 *μ*L of H_2_O_2_ (1 M). The reaction was incubated for 30 min. After 30 min PicoGreen® DNA dye was added and the fluorescence was read (excitation at 480 nm/emission at 520 nm). The genoprotective effect was considered present when the absorbance was lower than the positive control group [19].

### 2.6. Cytotoxic Assays

Two assays were used to test the potential cytotoxic effect of barbatimão on keratinocytes and fibroblasts: trypan dye exclusion assay and cf-DNA PicoGreen assay. The cf-DNA PicoGreen assay is based on the presence of double-stranded DNA fragments that are released when cells die as the cytoplasmic and nuclear membrane rupture. The cellular mortality rate is quantified by supernatant cell-free DNA (cf-DNA) levels using PicoGreen dye®, a fluorescent marker of double-stranded DNA fragments. Therefore, high cf-DNA levels indicate high mortality rate in the cell culture [19]. This assay was performed using Invitrogen Quant-IT kit following manufacturer's instructions. Ten microliters of cell culture supernatants were collected and added in a 96-well black plate together with reagents from Quant-iTTM PicoGreen and diluted in Tris-EDTA (TE) buffer (10mM Tris–HCl, 1 mM EDTA, pH 7.5). Then, 100 *μ*L of the PicoGreen dye diluted 1:200 in TE buffer was added to the microplate to make a final volume of 200 *μ*L per well. Following incubation in the dark for 10 min at room temperature, the fluorescent signal of the samples was measured at 480 nm excitation and 520 nm emission at room temperature using SpectraMax M2/M2e Multimode Plate Reader, Molecular Devices' equipment. Elevated cf-DNA levels indicated high cellular mortality.

### 2.7. Reactive Oxygen Species (ROS) Assay

As barbatimão is rich in antioxidant molecules, ROS levels of cell cultures were quantified using a 2,7 dichlorofluorescein diacetate (DCFH- DA) ROS production assay. DCFH-DA is hydrolysed by intracellular esterase enzymes to DCFH, which is trapped within the cell, and the nonfluorescent molecule is then oxidized with fluorescent dichlorofluorescein (DCF) using cellular oxidants. Therefore, to quantify ROS levels, cell cultures were treated with DCF-DA (10 mM) for 60 min at 37°C. The fluorescence was measured at an excitation of 488 nm and an emission of 525 nm, and the results were obtained as pmole/mL of DCF production from 2,7 dichlorofluorescin in reaction with ROS molecules present in the samples [27]. After the data were obtained, results were converted to % of control group.

### 2.8. Immunoassay Tests

The levels of DNA 8-OHdG were used to quantify DNA damage and those of CASP 3 and 8 were measured using a Quantikine Human Kit according to the manufacturer's instructions. All reagents and working standards were prepared and the excess microplate strips were removed before adding 50 *μ*L of the assay diluent RD1W to each well. 100 *μ*L of standard control for the sample was added per well, after which the well was covered with an adhesive strip and incubated for 1.5 h at room temperature. Each well was subsequently aspirated and washed twice, for a total of three washes. The antiserum of each molecule analyzed here was added to each well and covered with a new adhesive strip before being incubated for 30 min at room temperature. The aspiration/wash step was repeated, and the molecule-1 conjugate (100 *μ*L) was added to each well and incubated for 30 min at room temperature. The aspiration/wash step was repeated before adding 100 *μ*L of substrate solution to each well, followed by incubation at room temperature for an additional 20 min. Finally, 50 *μ*L of stop solution was added to each well and the optical density was determined within 30 min using a microplate reader set to 450 nm.

### 2.9. mRNA Expression Analysis by Quantitative QT-PCR Assay

Gene expressions of* BAX* and* Bcl-2* were analyzed in cells exposed to barbatimão extract. Total RNA was extracted using Trizol, following the manufacturer's instructions (Ludwig-Biotec, RS, Brazil). The extracted RNA was measured by a Thermo Scientific NanoDrop™ 1000 Spectrophotometer. To perform reverse transcription, 1 *μ*g/mL RNA was added to the samples with 0.2 *μ*L of DNAase at 37°C for 5 min, followed by heating at 65°C for 10 min. The cDNA was generated with 1 *μ*L of Iscript cDNA and 4 *μ*L of Mix Iscript. The next steps of the reaction were 5°C for 10 min, 25°C for 5 min, and 85°C for 5 min, with a final incubation at 5°C for 60 min. The qRT-PCR was performed in the Rotor-Gene Q 5plex HRM System (QIAGEN biotechnology, NW, Germany) using QuantiFast SYBR® Green PCR Master Mix, starting with a temperature of 95°C for 3 min followed by 40 cycles of 95°C for 10 s, 60°C for 30 s and a melt curve of 60°C to 90°C in 0.5°C increments for 5 s. The reactions of each sample were made in triplicate, using 1 *μ*M of each primer and with 2× QuantiFast SYBR® Green PCR Master Mix; the final volume was 20 *μ*L. The beta-actin gene sense (5′TGTGGATCAGCAAGCAGGAGTA3′) antisense (5′TGCGCAAGTTAGGTTTTGTCA3′) was used as a housekeeping gene:,*BAX* gene sense (5′CCCTTTTCTACTTTGCCAGCAA3′) antisense (5′CCCGGAGGAAGTCCAATGT3′),* BCL-2* gene sense (5′GAGGATTGTGGCCTTCTTTGAGT3′) antisense (5′AGTCATCCACAGGGCGATGT3′) [23].

### 2.10. Statistical Analysis

Statistical tests were performed using Graph Pad Prism Software. Results of all experiments were replicated three times with, at least, five repetitions of each treatment. Data from repetitions were evaluated before statistical analysis and normalized by % of control group. Therefore, results were expressed as % mean ± SD (standard deviation) of the control group. This procedure is broadly used in the in vitro protocols (Antonieli et al., 2017) to allow comparison between results obtained on different days, by different tests, and by different laboratories. The upper and lower values of 2-SD range were considered outliers and excluded of the analysis, because generally these outliers generate relative SD > 10% indicating presence of some experimental imprecision. Treatments were statistically compared by one-way analysis of variance (ANOVA), followed by Tukey* post hoc* test. In results showed in Figures different letters identified statistical differences (p ≤ 0.05) among treatments.

## 3. Results

Details of the barbatimão tree, bark, extract preparation samples, and chemical characterization are presented in Figure 2. Barbatimão hydroalcoholic extract presented higher levels (mg/g) of gallic acid (12.48±0.05), caffeic acid (8.06±0.02), quercetin (8.16±0.04), catechin (5.93±0.01), and rutin (4.71±0.01) (Figure 2). Low levels of kaempferol and other bioactive molecules were also detected (Figure 2).

Initially potential genoprotective capacity of barbatimão extract at different concentrations was determined and results are presented in Figure 3(a). In this assay, the control group was compounded by a solution containing isolated dsDNA plus H_2_O_2_ that caused extensive DNA fragmentation. When barbatimão extract was added in this solution an inverse dose-dependent action on dsDNA was observed. In this case, barbatimão at lower concentration (0.012 mg/mL) presented higher protective effect against H_2_O_2_ dsDNA fragmentation than more elevated concentrations. Concentrations near to barbatimão therapeutic doses used to healing wound showed some dsDNA protection, but this effect was less intense than that observed in the other lower concentrations.

The first* in vitro* protocol performed here using keratinocytes (Figure 3(b)) and fibroblast (Figure 3(c)) cells evaluated potential barbatimão effect on viability of these cells using the same range of concentrations evaluated by GEMO assay (Figure 3(a)). Barbatimão did not present a cytotoxic effect on keratinocytes and fibroblasts when compared to control group that did not receive any additional treatment numerically represented by 0 value in the graphic. On the contrary, cell culture barbatimão extract supplementation increased viability of both cell lines when compared to no-treated control group (0). However, this effect was higher in cultures supplemented with intermediary concentrations (Keratinocytes: 0.099-0.49 mg/mL; fibroblasts: 0.099-0.99 mg/mL).

Two barbatimão concentrations (0.49 and 0.99 mg/mL) near to those found in commercial preparations were used to perform analysis of genotoxicity in keratinocyte and fibroblast 24 h cell cultures. In cells exposed to these concentrations, DNA damage quantified by DNA 8-OHdG levels,* BAX*/*Bcl-2* gene expression ratio, and CASP 3 and 8 levels were quantified. In both barbatimão concentrations there was a significant decrease in ROS levels when 24 h keratinocyte cultures were exposed to 0.49 mg/mL (87.2±2.5% of control group) and 0.99 mg/mL (74.3±3.0% of control group). Similar results were also found in fibroblasts, where barbatimão extract at 0.49 mg/mL reduced ROS levels to 82.4±3.0% of control group and at 0.99 mg/mL reduced ROS levels to 69.5±3.0% of control group (*p* ≤ 0.001).

**Figure 4:**
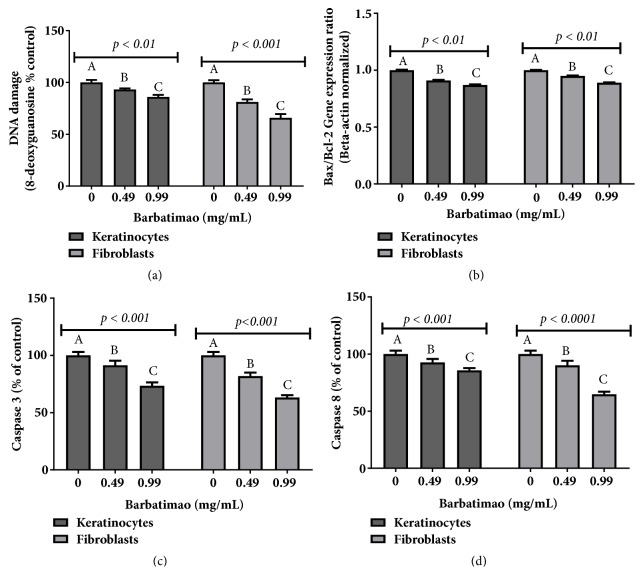
Comparison among genotoxic and apoptotic markers of keratinocytes and fibroblasts exposed to barbatimão hydroalcoholic extraction. (a) 8-OHdG levels; (b)* BAX*/*Bcl-2* gene expression ratio quantified by qRT-PCR, that indicates modulation of intrinsic apoptotic events; (c) caspase 3 protein levels; (d) caspase 8 protein levels. Data were compared by one-way analysis of variance (ANOVA) followed by a Tukey* post hoc* test. In each marker tested here, statistical differences at p ≤ 0.05 among 24 h cell cultures treatments were identified by different letters (A, B, C).

Barbatimão treated samples also showed a decrease in DNA damage evaluated by 8-OHdG levels (Figure 4(a)) in both 24 h cell cultures tested. Analysis of apoptotic markers showed a decrease in* BAX*/*Bcl-2* gene expression ratio (Figure 4(b)). Moreover, barbatimão decreased CASP 3 and 8 in both keratinocyte and fibroblast cell lines (Figures 4(c) and 4(d)). However, the genoprotective and caspase lowering effect was more pronounced in fibroblasts than in keratinocytes.

**Figure 5:**
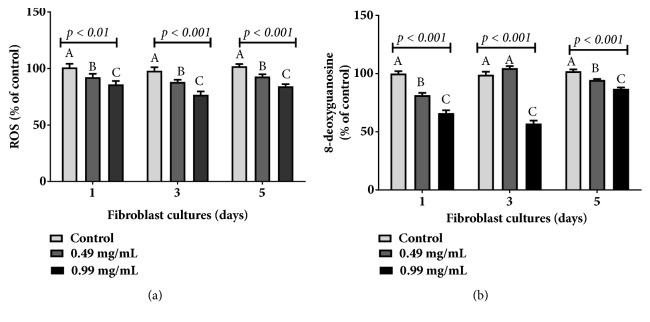
Modulation of oxidative markers on three different cell culture days of keratinocytes and fibroblasts exposed to two different hydroalcoholic barbatimão extracts to evaluate potential chronic genotoxicity of this extract on cells. (a) Reactive oxygen species (ROS) levels; (b) 8-OHdG levels. Data were compared by one-way analysis of variance (ANOVA) followed by a Tukey post hoc test. In each marker tested here, statistical differences at p ≤ 0.05 among 24 h cell cultures treatments were identified by different letters (A, B, C).

Considering that the effect of barbatimão could be transient and cause subsequent triggering of an increase in ROS levels and DNA damage in older cultures, a complementary analysis was performed in fibroblasts in order to evaluate the effect of barbatimão on 1, 3, and 5 day cell cultures. As seen in Figure 5, barbatimão maintained its antioxidant and genoprotective effect in cultures of several days old. However, this result was more pronounced in 72 h cell cultures exposed to 0.99 mg/mL concentration of barbatimão extract.

## 4. Discussion

The present investigation evaluated potential genotoxic effects of barbatimão, a Brazilian plant used traditionally for wound healing. Most results obtained from the* in vitro* protocol using keratinocyte and fibroblasts indicated noncytotoxic and genoprotective effects of barbatimão extract. The genoprotective effect was estimated by a noncellular assay, quantification of 8-deoxyguanosine levels, and gene and proteins involving in apoptosis processes in both cell lines. These results could be considered relevant since use of phytotherapeutics from traditional medicine has been considered a part of healthcare by the World Health Organization (WHO) since 2002. Barbatimão has been used as an astringent, antihemorrhagic, and antidiarrheal as well as a treatment for scurvy, genitourinary disorders, and lower airways conditions [2].

The Brazilian Official Pharmacopoeia [28] estimated that the wound-healing effect of barbatimão could be associated with the high tannin concentration in its dried bark (8%). In fact, most bioactive molecules quantified in the barbatimão extract used here were previously described in the literature, such as gallic acid [29] and catechins [3]. It is important to mention that other bioactive molecules of barbatimão such as quercetin, rutin, and kaempferol have been identified and quantified in this study and have not been described in previous chemical analyses of barbatimão. Quercetin and rutin, also called quercetin-3-O-rutinoside, are flavonoids found in several fruits and vegetables including citrus and onion. Studies on quercetin have described interesting biological effects including cell protection against UV irradiation and skin regeneration in wound healing [30].

Due to its commercial use, it is important to clarify the potential genotoxic activity of barbatimão in human cells. There are relatively few studies that evaluate potential genotoxic effect of barbatimão, leading to the controversy over the safety of this traditional medicine. Costa et al. [15] evaluated potential micronucleus induction in mice orally treated with proanthocyanidin polymer-rich fraction (F2) of the stem bark of barbatimão and did not find a genotoxic effect of these molecules. Moreover, a study involving potential somatic mutation and recombination analysis (SMART test) and chromosome damage in germ cell lines of fruit flies exposed to barbatimão bark extract did not find genotoxic effect of this plant [31]. However, when Vilar et al. [32] evaluated lyophilized solution produced from barbatimão stem bark, results indicated some cytotoxic and genotoxic effect using SOS-Inductest in* E. coli* prokaryotic model. Therefore, the authors considered it important that further* in vitro *and* in vivo* studies are performed to clarify these potentially negative results.

In this context, it is plausible to consider that triggering healing with barbatimão extract could cause some undesirable chromosomal instability associated with procarcinogenic processes. Initially, we obtained data from the GEMO assay that indicated the genoprotective activity of barbatimão extract. This validated assay was developed by Cadoná et al. [19] due to the necessity of identifying the genoprotective and genotoxic capacity of plant extracts that are generally analyzed in* in vitro* cell cultures, which are very expensive, and present some influence on testing response due to differential biochemical and molecular specifications of each cell line. Therefore, results obtained from the GEMO assay are fast and inexpensive, involving double-strand DNA (dsDNA) damage caused by H_2_O_2_ exposure that was completely reverted in the presence of barbatimão, mainly in lower concentrations of the extract.

Subsequent analysis confirmed no genotoxic effect of barbatimão by analysis of 8-OHdG levels, which decreased in the presence of this extract. This molecule is considered a biomarker of oxidative stress and oxidative DNA damage found both in physiological fluids and cells and is frequently used as a marker of exposure to oxidants as well as a potential marker of some chronic nontransmissible disease progression [33, 34]. In the presence of 0.49 and 0.99 mg/mL barbatimão 8-OHdG levels were shown to decrease, suggesting that barbatimão extract could act as genoprotective compound.

Barbatimão extract also decreased* BAX*/*Bcl-2* gene expression ratio, and CASP 3 and 8 protein levels. These markers are related mainly to intrinsic apoptosis triggered via accumulation of DNA lesions from genotoxic compound exposure [33].

## 5. Conclusions

Despite methodological limitations related to* in vitro *studies, our data corroborate results found in rats and fruit flies with respect to lack of genotoxicity and safety. Moreover, the results described here suggest that barbatimão could present a genoprotective and antiapoptotic effect on human keratinocytes and fibroblasts.

## Data Availability

The data used to support the findings of this study are included in the article.
